# Genetic redundancy in the catabolism of methylated amines in the yeast *Scheffersomyces stipitis*

**DOI:** 10.1007/s10482-017-0963-y

**Published:** 2017-10-30

**Authors:** Tomas Linder

**Affiliations:** Department of Molecular Sciences, Swedish University of Agricultural Sciences, Box 7015, 750 07 Uppsala, Sweden

**Keywords:** Amine, Metabolism, Reverse genetics, Yeast

## Abstract

The catabolism of choline as a source of nitrogen in budding yeasts is thought to proceed via the intermediates trimethylamine, dimethylamine and methylamine before the release of ammonia. The present study investigated the utilisation of choline and its downstream intermediates as nitrogen sources in the yeast *Scheffersomyces stipitis* using a reverse genetics approach. Six genes (*AMO1*, *AMO2*, *SFA1*, *FGH1*, *PICST_49761*, *PICST_63000*) that have previously been predicted to be directly or indirectly involved in the catabolism of methylated amines were individually deleted. The growth of each deletion mutant was assayed on minimal media with methylamine, dimethylamine, trimethylamine or choline as the sole nitrogen source. The two amine oxidase-encoding genes *AMO1* and *AMO2* appeared to be functionally redundant for growth on methylated amines as both deletion mutants displayed growth on all nitrogen sources tested. However, deletion of *AMO1* resulted in a pronounced growth lag on all four methylated amines while deletion of *AMO2* only caused a growth lag when methylamine was the sole nitrogen source. The glutathione-dependent formaldehyde dehydrogenase-encoding gene *SFA1* was found to be absolutely essential for growth on all methylated amines tested while deletion of the *S*-formylglutathione hydrolase gene *FGH1* caused a pronounced growth lag on dimethylamine, trimethylamine and choline. The putative cytochrome P450 monooxygenase-encoding genes *PICST_49761* and *PICST_63000* were considered likely candidates for demethylation of di- and trimethylamine but produced no discernable phenotype on any of the tested nitrogen sources when deleted. This study revealed notable instances of genetic redundancies in the choline catabolic pathway, which are discussed.

**Electronic supplementary material:**

The online version of this article (doi:10.1007/s10482-017-0963-y) contains supplementary material, which is available to authorized users.

## Introduction

Amines are organic nitrogen compounds common in the environment and consequently most microorganisms have evolved pathways to assimilate amines as a source of metabolic nitrogen. The ability to assimilate amines as a nitrogen source is nearly universal among budding yeasts (phylum *Ascomycota*, sub-phylum *Saccharomycotina*) but does not occur in the common model system *Saccharomyces cerevisiae* (van der Walt 1962; van Dijken and Bos 1981; Linder 2014). Due to the limited research hitherto conducted on non-*Sa. cerevisiae* yeasts, our understanding of the genetics governing the assimilation of amines in budding yeasts remains rudimentary.

Previous biochemical studies on budding yeasts capable of assimilating amines have shown that the de-amination of primary amines (RCH_2_NH_2_) is catalysed by copper-containing amine oxidases (EC 1.4.3.6) to release ammonia, hydrogen peroxide and the corresponding alkylaldehyde (RCHO). Most budding yeasts appear to possess two types of amine oxidases, which are commonly referred to as methylamine oxidase and benzylamine oxidase, respectively (Haywood and Large 1981; Green et al. 1982). Methylamine oxidase (encoded by the *AMO1* gene) appears to have higher affinity towards short-chain aliphatic amines while benzylamine oxidase (encoded by the *AMO2* gene) has higher affinity towards amines with longer and bulkier side-chains (Haywood and Large 1981). The Amo1 methylamine oxidase contains an N-terminal type 2 peroxisomal targeting signal (PTS2) composed of the nonapeptide motif RLXXXXX^H^/_Q_L and localises to the peroxisome (Zwart et al. 1980; Faber et al. 1994) while the Amo2 benzylamine oxidase is thought to be localised to the cytosol.

**Fig. 1:**
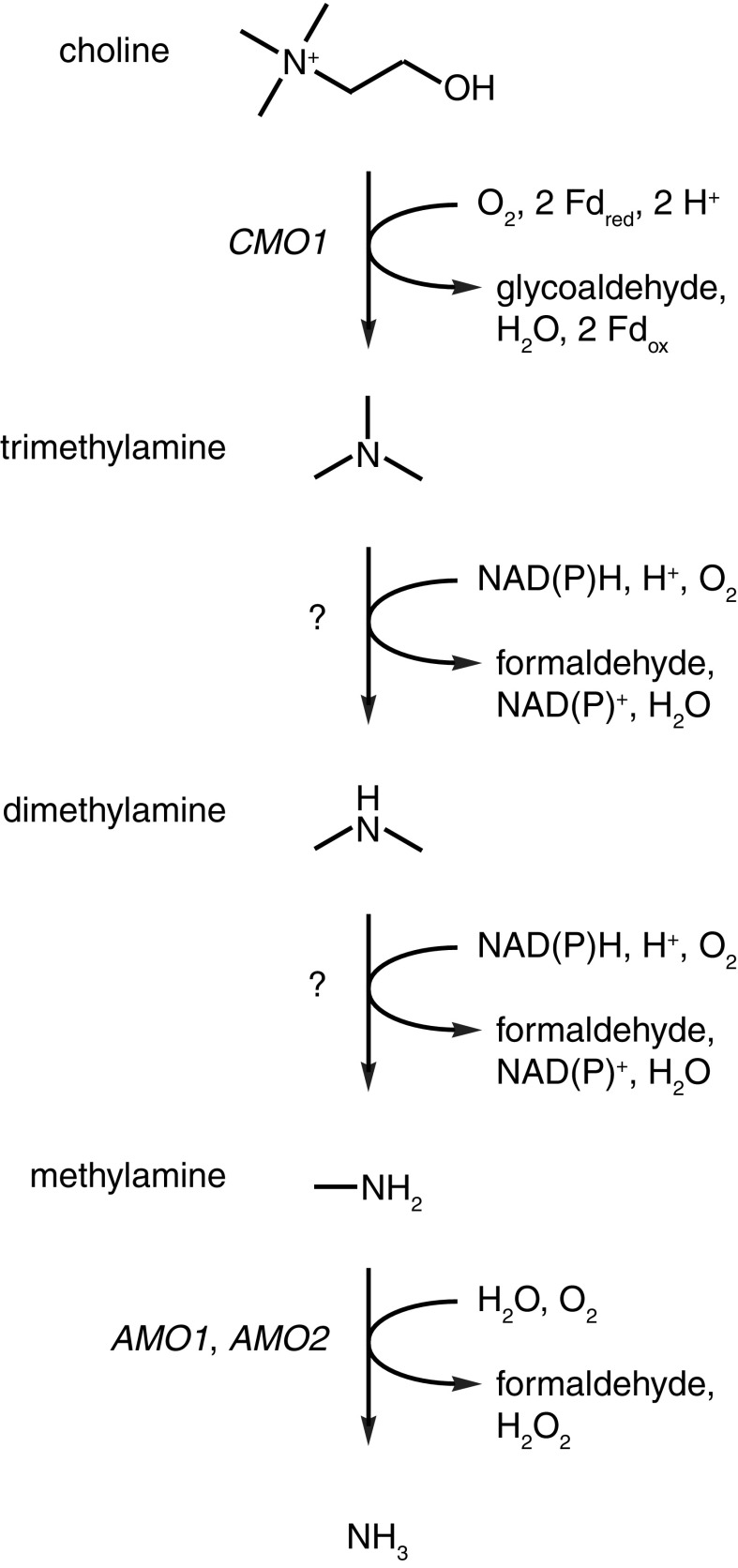
The proposed pathway for choline assimilation in budding yeasts

Secondary [(RCH_2_)_2_NH] and tertiary [(RCH_2_)_3_N] amines can also be assimilated by some yeasts (van Dijken and Bos 1981; Linder 2014) but the identity of the enzymes involved in the de-alkylation of these substrates into primary amines have yet to be established. Choline is one of the few quaternary (tetra-alkylated) amines that is known to be de-alkylated and subsequently assimilated by budding yeasts (van Dijken and Bos 1981; Linder 2014). The catabolism of choline is thought to involve four sequential de-alkylation steps via the intermediates trimethylamine, dimethylamine and methylamine before ammonia is released by amine oxidase (Zwart et al. 1980, 1983; Fig. 1). Understanding of the genetics surrounding this pathway remains incomplete. A putative choline monooxygenase encoded by the *CMO1* gene is thought to be responsible for the first de-alkylation step to produce trimethylamine and glycolaldehyde (Mori et al. 1988; Linder 2014). The yeast Cmo1 protein has not yet been biochemically characterised but the homologous plant protein (which converts choline into betaine aldehyde in the chloroplast) requires oxygen and ferredoxin (Brouquisse et al. 1989). Biochemical studies of the demethylation of di- and trimethylamine indicate that two distinct enzyme activities are involved, which have characteristics typical of cytochrome P450 monooxygenases (Green and Large 1983, 1984; Fattakhova et al. 1991). The final demethylation step involves one or both amine oxidases (Haywood and Large 1981).

The present study set out to study the genetics surrounding the catabolism of choline and focused on six genes thought to be directly involved in either the amine de-alkylation steps or linked reactions such as detoxification of the significant amounts of formaldehyde generated by each demethylation reaction. The yeast *Scheffersomyces stipitis* was selected as a model system for this study as it can assimilate all the predicted intermediates of the choline pathway (Linder 2014).

## Materials and methods

### *Sc. stipitis* integration constructs

Targeting cassettes for *AMO1* (*PICST_55523*), *AMO2* (*PICST_83878*), *SFA1* (*PICST_29252*), *FGH1* (*PICST_65460*), *PICST_49761* and *PICST_63000* were synthesised *de novo* by GenScript (NJ, USA) and inserted into either EcoRI/HindIII-cut or EcoRI/StuI-cut pUC57 (GenBank accession Y14837; Fig. S1a). Each targeting cassette consisted of 500 bp sequence identical to the intergenic region immediately downstream (3′ IGR) of the gene to be deleted (“*PICST_xxxxx*”) followed by 500 bp sequence immediately upstream (5′ IGR) of the sequence to be deleted (Fig. S1b). The 3′ and 5′ IGR sequences in each targeting cassette were separated by a SwaI recognition site. Each targeting cassette was followed by a short polylinker to enable insertion of a selection marker. The full-length *HIS3* gene was amplified from *Debaryomyces hansenii* CBS 767 genomic DNA with primers DhHIS3 fwd (5′ GCG CGC GGA TCC TTT CAC CAG ATG GGA TCT AAT 3′) and DhHIS3 rev (5′ GCG CGC CTG CAG GCG CGC CAG TCG TAA TGT TTA TAG AAG A 3′), digested with BamHI and PstI and inserted into each targeting construct, which had been digested with the appropriate enzymes to produce ends compatible with BamHI (either BamHI or BglII) and PstI (PstI or SbfI). The restriction sites used to insert the *DhHIS3* gene into each targeting plasmid are indicated in Fig. S1a. Prior to transformation, the integration plasmids (Fig. S1c) were digested with SwaI to produce linearised integration constructs (Fig. S1d), which were then purified into sterile water using the QIAquick PCR purification kit (Qiagen).

### Yeast transformation

The transgenic yeast strains used in this study are listed in Table 1. The transformation methodology has been described previously (Linder 2014). The *Sc. stipitis* SF1 strain, which lacks a functional non-homologous end-joining DNA repair pathway (Maassen et al. 2008), was kindly provided by Prof Ulrich Klinner (Aachen University, Germany). Correct chromosomal integration and the deletion of each targeted gene (“*PICST_xxxxx*”) was confirmed by PCR analysis of purified genomic DNA from each deletion strain (Fig. S2) using the primers listed in Table S1.

**Table 1:** Genetically modified *Sc. stipitis* strains used in this study

Strain number	Relevant genotype	Source
SF1	*his3*-*1 trp5*-*10 YKU80*::*ScTRP5*	U. Klinner (Maassen et al. 2008)
TLSS001	*HIS3 trp5*-*10 YKU80*::*ScTRP5*	Linder (2014)
TLSS005	*his3*-*1 trp5*-*10 YKU80*::*ScTRP5 PICST_55523*::pUC57-*DhHIS3*	This study
TLSS006	*his3*-*1 trp5*-*10 YKU80*::*ScTRP5 PICST_83878*::pUC57-*DhHIS3*	This study
TLSS007	*his3*-*1 trp5*-*10 YKU80*::*ScTRP5 PICST_29252*::pUC57-*DhHIS3*	This study
TLSS008	*his3*-*1 trp5*-*10 YKU80*::*ScTRP5 PICST_65460*::pUC57-*DhHIS3*	This study
TLSS009	*his3*-*1 trp5*-*10 YKU80*::*ScTRP5 PICST_49761*::pUC57-*DhHIS3*	This study
TLSS010	*his3*-*1 trp5*-*10 YKU80*::*ScTRP5 PICST_63000*::pUC57-*DhHIS3*	This study

### Nitrogen utilisation assays

Sodium L-glutamate and the hydrochloride salts of methylamine, dimethylamine, trimethylamine and choline were purchased from Sigma Aldrich. A reduced sulfur/nitrogen-limited glucose medium (RSNLD) was used for assaying growth on individual amines. RSNLD medium is composed of 1.7 g Difco yeast nitrogen base without amino acids or ammonium sulfate l^−1^ (Becton, Dickinson and Company) and 20 g glucose l^−1^. Prior to the nitrogen utilisation assay, individual yeast strains were pre-cultured in 3 ml minimal glucose medium (MMD) consisting of 6.7 g Difco yeast nitrogen base without amino acids l^−1^ (Becton, Dickinson and Company) and 20 g glucose l^−1^. Pre-cultures were washed twice in RSNLD before being re-suspended in 2.97 ml RSNLD to a final OD_600_ of 0.005 in a 50-ml tube. Individual nitrogen sources were added as 30 μl of a 1 M stock solution, making a final concentration of 10 mM. A non-supplemented sample with 30 μl deionised water was used as a control. Chloramphenicol (final concentration 15 mg l^−1^) was included to prevent bacterial contamination. Samples were incubated at 30 °C in a rotary shaker set to 200 rpm with OD_600_ measurements after 6, 12 and 18 days. OD_600_ measurements were carried out with a 1 cm path length using an Ultrospec 1100 pro spectrophotometer (GE Healthcare). Each growth assay was performed in triplicate with each biological replicate using a separate pre-culture. The yeast strain *Ogataea parapolymorpha* CBS 11895 was purchased from Centraalbureau voor Schimmelcultures (Utrecht, the Netherlands).

### Sequence alignment and phylogenetic analysis

All BLASTP and TBLASTN searches applied an expect value cut-off of 10^−5^ with the low-complexity region filter enabled. Protein sequences were aligned in MAFFT (Katoh et al. 2005; http://mafft.cbrc.jp/alignment/server/index.html). Selection of sequence positions suitable for phylogenetic analysis was carried out in GBlocks (Castresana 2000; http://molevol.ibmb.csic.es/Gblocks_server/). The resulting amino acid positions were then used to construct a neighbour-joining tree in MEGA v. 6 (Tamura et al. 2013) using a JTT amino acid substitution model. Branch support was tested using 10,000 bootstrap replicates. Any nodes with bootstrap values equal or less than 50 were collapsed. The consensus trees were visualised in FigTree v.1.0 (http://tree.bio.ed.ac.uk/software/figtree/).

## Results and discussion

Previous studies on the catabolism of choline and its putative downstream intermediates in budding yeasts have mainly employed biochemical and cell imaging methods (for example Zwart et al. 1980; Haywood and Large 1981; Zwart et al. 1983). Parallel analysis of gene deletion phenotypes under specific growth conditions (phenomics) is a useful tool for the identification of functional redundancies and genetic associations. Measuring cell growth in batch culture is particularly informative as it is possible to resolve the individual dynamic components of the typical sigmoidal cellular growth curve such as growth lag, growth rate and growth efficiency (Warringer et al. 2003). Very little reverse genetics have been done in *Sc. stipitis* to date due to a scarcity of selection markers and the low targeting frequency of integration constructs due to the domination of the non-homologous end-joining (NHEJ) pathway over homologous recombination. However, the development of a *Sc. stipitis* strain auxotrophic for histidine that also lacks a functional NHEJ pathway through deletion of the *YKU80* gene has now enabled reverse genetic investigations in this yeast (Maassen et al. 2008). Targeting cassettes for each of the six genes to be deleted in this study (*AMO1*, *AMO2*, *SFA1*, *FGH1*, *PICST_49761*, *PICST_63000*) were synthesised *de novo* to contain 500 bp of flanking sequences adjacent to the deleted region (Fig. S1a). The *D. hansenii HIS3* gene was inserted into each integration construct to enable selection for positive transformants on minimal medium lacking histidine (Fig. S1b-c). A *Sc. stipitis Δyku80* strain with a regenerated endogenous *HIS3* locus (Linder 2014) was used as a wildtype control in all growth assays.

**Fig. 2:**
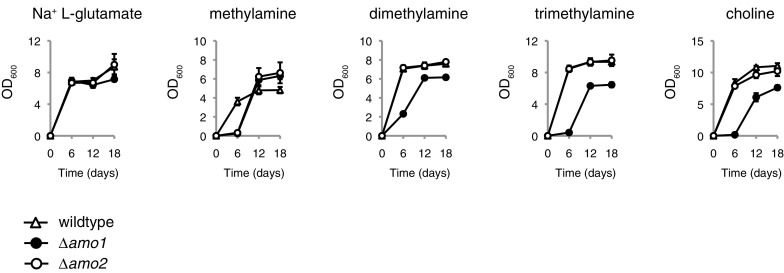
The requirement for amine oxidase genes *AMO1* and *AMO2* for the utilisation of methylated amines as sole nitrogen sources. *Sc. stipitis* strains TLSS001 (wildtype control), TLSS005 (*Δamo1*) and TLSS006 (*Δamo2*) were cultured in 3 ml RSNLD medium supplemented with 10 mM of the indicated nitrogen source (initial OD_600_ 0.005). Samples were incubated in a shaker set at 30 °C, 200 r.p.m., and OD_600_ was measured after 6, 12 and 18 days. Growth assays were performed in triplicate with error bars indicating one standard deviation

The first two genes to be investigated were the two amine oxidase-encoding genes *AMO1* and *AMO2*. Each deletion mutant was cultivated in nitrogen-limited medium supplemented with either sodium l-glutamate, methylamine, dimethylamine, trimethylamine or choline. Growth was monitored every 6 days up until 18 days after initiation of the growth assay (Fig. 2). Both mutants displayed strong growth on sodium l-glutamate equivalent to the wildtype control. Interestingly both mutants displayed an identical growth lag on methylamine with strong growth detectable only after the 12-day time-point. In addition, both mutant strains displayed notably higher cell densities at day 12 and 18 on methylamine compared to the wildtype control. The reason for this effect was not immediately obvious but one possibility could be that the initial lag phase indirectly enabled more efficient energy metabolism once growth was initiated. The *Δamo2* strain was indistinguishable from the wildtype control on dimethylamine, trimethylamine and choline, which indicated that *AMO2* was entirely dispensable for normal growth on these substrates. The *Δamo1* strain displayed a characteristic growth lag on dimethylamine, trimethylamine and choline, which indicated that *AMO1* was only partially dispensable for growth on these substrates.

It was notable that the *Δamo2* strain had no obvious growth defect on dimethylamine, trimethylamine or choline yet displayed a pronounced lag in growth on methylamine, which is a down-stream intermediate of the three other amines. One possibility is that when methylamine is supplied as an external nitrogen source, the activities of *AMO1* and *AMO2* must cooperate to achieve maximum growth while *AMO2* does not play a significant role in the deamination of methylamine generated intracellularly through demethylation of di- and trimethylamine.

**Fig. 3:**
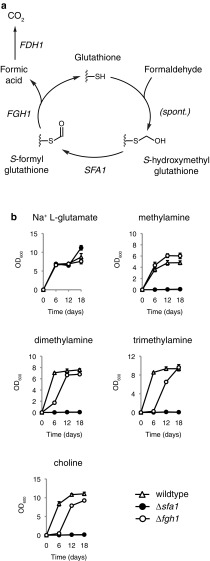
The requirement for the glutathione-dependent formaldehyde dehydrogenase gene *SFA1* and the *S*-formylglutathione hydrolase gene *FGH1* for the utilisation of methylated amines as sole nitrogen sources. **a** A simplified overview of the glutathione-dependent formaldehyde detoxification pathway. Only the thiol group of glutathione (-SH) is shown. **b** *Sc. stipitis* strains TLSS001 (wildtype control), TLSS007 (*Δsfa1*) and TLSS008 (*Δfgh1*) were cultured in 3 ml RSNLD medium supplemented with 10 mM of the indicated nitrogen source (initial OD_600_ 0.005). Samples were incubated in a shaker set at 30 °C, 200 r.p.m., and OD_600_ was measured after 6, 12 and 18 days. Growth assays were performed in triplicate with error bars indicating one standard deviation

Each of the three demethylation steps in the choline assimilation pathway produces an equimolar amount of formaldehyde to the amount of methylated amine (Fig. 1). The assimilation of methylated amines therefore generates significant amounts of highly reactive formaldehyde that must be appropriately metabolised by the cell to ensure viability. The principal pathway for the detoxification of formaldehyde in yeast is the cyclic glutathione-dependent formaldehyde oxidation pathway (Fig. 3a). Formaldehyde enters the pathway through a non-enzymatic reaction with glutathione to form *S*-hydroxymethylglutathione. *S*-hydroxymethylglutathione is then converted into *S*-formylglutathione by glutathione-dependent formaldehyde dehydrogenase, which is encoded by the *SFA1* gene in yeast (Sasnauskas et al. 1992). Glutathione is then regenerated through the action of *S*-formylglutathione hydrolase (encoded by the *FGH1* gene) to release formic acid (Degrassi et al. 1999; Yurimoto et al. 2003). Formic acid is subsequently oxidised into carbon dioxide by formate dehydrogenase, which is encoded by the *FDH1* gene (Allen and Holbrook 1995). The deletion of either *SFA1* or *FGH1* was therefore expected to cause rapid glutathione depletion and ultimately cell death under conditions of high levels of formaldehyde production, such as cultivation on medium where methylated amines were the sole nitrogen source.

No obvious growth defect with sodium l-glutamate as nitrogen source was observed upon deletion of either *SFA1* or *FGH1* in *Sc. stipitis* (Fig. 3b). As expected, the *Δsfa1* strain did not show any detectable growth when any of the methylated amines were the sole nitrogen source. This is in contrast with previous work in the methylotrophic yeast *Ogataea boidinii* where the *Δsfa1* cells displayed weak but detectable growth on both methylamine and choline (Lee et al. 2002). One possibility is that *O. boidinii* possesses a second functionally redundant enzyme capable of converting *S*-hydroxymethylglutathione into *S*-formylglutathione.

Notably the *Δfgh1* strain showed significant growth on all methylated amines and in the case of methylamine the *Δfgh1* strain displayed noticeably higher cell densities than the wildtype control. However, the *Δfgh1* strain displayed a pronounced growth lag on dimethylamine, trimethylamine and choline reminiscent of the growth dynamics observed with the *Δamo1* strain (Fig. 2). Direct comparison of the growth curves for the *Δfgh1* and *Δamo1* strains showed a striking overlap in growth dynamics (Fig. S3), which could indicate a genetic link between the two genes. One possibility is that Amo1-dependent demethylation of methylamine derived from dimethylamine, trimethylamine and choline in *Sc. stipitis* requires Fgh1 while Amo2-dependent demethylation does not. In *O. boidinii* the Fgh1 protein has been shown to localise both in peroxisomes and the cytosol (Yurimoto et al. 2003). Assuming that the *Sc. stipitis* Fgh1 protein displays the same localisation pattern as its *O. boidinii* ortholog, the Fgh1 protein would available to both amine oxidase isoenzymes. The fact that *SFA1* is essential for growth on methylated amines in *Sc. stipitis* but *FGH1* is not would suggest there are additional pathways in this yeast for the regeneration of glutathione from *S*-formylglutathione. Previous studies of the *FGH1* gene in *O. boidinii* have shown that deletion of *FGH1* in this yeast results in detectable but retarded growth on both methylamine and choline (Yurimoto et al. 2003), which would suggest the existence of a functionally redundant enzyme in this yeast as well.

The enzymes responsible for demethylation of di- and trimethylamine in budding yeasts remain to be identified (Fig. 1). Previous biochemical studies have suggested that cytochrome P450 monooxygenases may be responsible for these reactions (Green and Large 1983, 1984; Fattakhova et al. 1991). A survey of *Sc. stipitis* genes encoding proteins belonging to the cytochrome P450 (CYP) monooxygenase family was therefore conducted. A total of ten genes in the *Sc. stipitis* genome have been assigned to the CYP superfamily (Chen et al. 2014; Fig. S4). Three of the *Sc. stipitis* CYP genes appeared to be the orthologs of the *Sa. cerevisiae* genes *DIT2* (*N*-formyltyrosine oxidase, family CYP56), *ERG5* (C-22 sterol desaturase, family CYP61) and *ERG11* (lanosterol 14α-demethylase, family CYP51), respectively. The genes *ERG5* and *ERG11* are involved in the biosynthesis of the essential membrane steroid ergosterol (Turi et al. 1991; Kelly et al. 1995) and both genes have so far been found in all sequenced yeast genomes. The *DIT2* gene is involved in the biosynthesis of dityrosine, which is a component of the spore cell wall (Briza et al. 1994). However, the *DIT2* gene does not appear to be universally conserved among budding yeasts (data not shown).

The remaining seven CYP-family genes lacked *Sa. cerevisiae* counterparts but five of them shared sequence similarity with *n*-alkane monooxygenases and fatty acid w-hydroxylases (*ALK*, family CYP52), which have been previously described in other yeasts (Sanglard and Loper 1989; Ohkuma et al. 1995; Huang et al. 2014). The two remaining *Sc. stipitis* CYP genes *PICST_49761* and *PICST_63000* have previously been assigned to CYP families CYP501 and CYP5217, respectively (Chen et al. 2014). Neither of these two CYP families have been biochemically characterised at the time of writing.

To avoid the necessity of deleting all seven CYP genes, a bioinformatic survey was made of genomes of other budding yeasts to identify species capable of assimilating multi-alkylated amines while simultaneously possessing a smaller repertoire of CYP-family genes than *Sc. stipitis*. The genome of the yeast *Ogataea parapolymorpha* was found to contain five CYP-family genes in total (Chen et al. 2014; Fig. S4) as well as a homolog of the *CMO1* gene (systematic gene name *HPODL_01912*), which predicted that this species should be able to assimilate choline and therefore di- and trimethylamine as well. *O. parapolymorpha* was therefore tested for its ability to use trimethylamine as a nitrogen source and was found to display strong growth (Fig. S5a). Phylogenetic analysis of the peptide sequences from the full CYP gene family complement of *O. parapolymorpha*, *Sa. cerevisiae* and *Sc. stipitis* identified two distinct clades of unknown function, which contained CYP-family proteins from both *O. parapolymorpha* and *Sc. stipitis* but none from *Sa. cerevisiae* (Fig. S5b). The first clade contained the gene products of the *O. parapolymorpha* and *Sc. stipitis* genes *HPODL_02874* and *PICST_63000*, respectively. The bootstrap support for this clade was moderate (68%) with the two proteins being 30% identical and 45% similar. A recent classification effort of fungal CYP genes placed the *HPODL_02874* and *PICST_63000* genes in separate CYP families CYP5223 and CYP5217, respectively (Chen et al. 2014). The second clade had strong bootstrap support (97%) and consisted of the gene products of *O. parapolymorpha* genes *HPODL_02307* and *HPODL_00882* as well as the gene product of the *Sc. stipitis* gene *PICST_49761*. *HPODL_02307* formed an internal node with *PICST_49761* with 83% bootstrap support and the protein sequences of the gene products were 37% identical and 54% similar. The *HPODL_02307* and *PICST_49761* genes are currently assigned to the CYP501 family while the *HPODL_00882* gene has been placed in the CYP504 family (Chen et al. 2014).

**Fig. 4:**
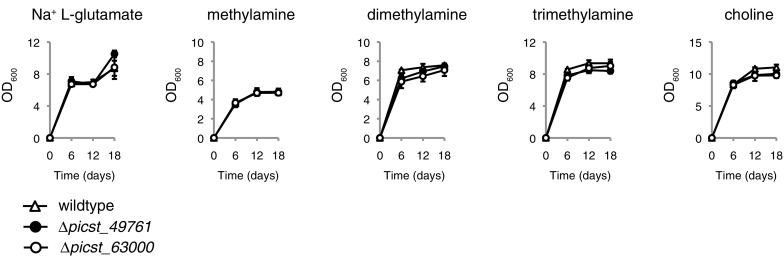
The requirement for the putative cytochrome P450 (CYP) monooxygenase genes *PICST_49761* and *PICST_63000* for the utilisation of methylated amines as sole nitrogen sources. *Sc. stipitis* strains TLSS001 (wildtype control), TLSS009 (*Δpicst_49761*) and TLSS010 (*Δpicst_63000*) were cultured in 3 ml RSNLD medium supplemented with 10 mM of the indicated nitrogen source (initial OD_600_ 0.005). Samples were incubated in a shaker set at 30 °C, 200 r.p.m., and OD_600_ was measured after 6, 12 and 18 days. Growth assays were performed in triplicate with error bars indicating one standard deviation

The assumption was made that if CYP-family enzymes were involved in the demethylation of di- and trimethylamine, these enzymes were expected to be shared between *Sc. stipitis* and *O. parapolymorpha* but lacking in *Sa. cerevisiae*. The only two *Sc. stipitis* CYP-encoding genes that satisfied this criterion were the two uncharacterised genes *PICST_49761* and *PICST_63000*. The two *Sc. stipitis* genes *PICST_49761* and *PICST_63000* were therefore deleted and tested for growth on different nitrogen sources. However, neither deletion mutant displayed any obvious growth defect on methylated amines compared to the wildtype control (Fig. 4). This suggests that *PICST_49761* and *PICST_63000* do not encode either of the CYP-family enzymes or CYP-like enzymatic activities thought to catalyse the demethylation of di- and trimethylamine. These results suggest that the enzymes responsible for the demethylation of di- and trimethylamines may in fact belong to another enzyme family with biochemical properties similar to that of CYP monooxygenases that had been reported previously (Green and Large 1983, 1984; Fattakhova et al. 1991). Another possibility is that a diversity of pathways exists in different yeasts for the demethylation of di- and trimethylamine, where one or more alternative enzyme families are responsible for these enzymatic steps in *Sc. stipitis* while CYP-family enzymes catalyse these reactions in the yeast species studied previously. A third possibility is that there is functional redundancy within the CYP superfamily so that the *Sc. stipitis* CYP52 family *ALK*-like monooxygenases also possess amine demethylation activity. This could be tested through sequential deletion of all seven members of CYP families CYP52, CYP501 and CYP5217 in *Sc. stipitis*. However, the current limitation in genetic tools for this *Sc. stipitis* makes this approach impractical at present.

In summary, this study demonstrates the substantial gap that remains in functional annotation of genomes from so-called non-conventional yeasts. The common baker’s yeast *Sa. cerevisiae* has long been the dominant system for the application of reverse genetics to the study of yeast metabolism. However, the limited number of nitrogen substrates assimilated by *Sa. cerevisiae* makes it unsuited to the study of poorly characterised nitrogen assimilation pathways (Large 1986; Linder 2014). This study focused specifically on the yeast choline catabolic pathway, which has previously been studied using predominantly biochemical methods (for example Green and Large 1983, 1984; Mori et al. 1988; Fattakhova et al. 1991). A complementary reverse genetics approach was used in this study and the resulting data can be condensed into three general observations.

**Fig. 5:**
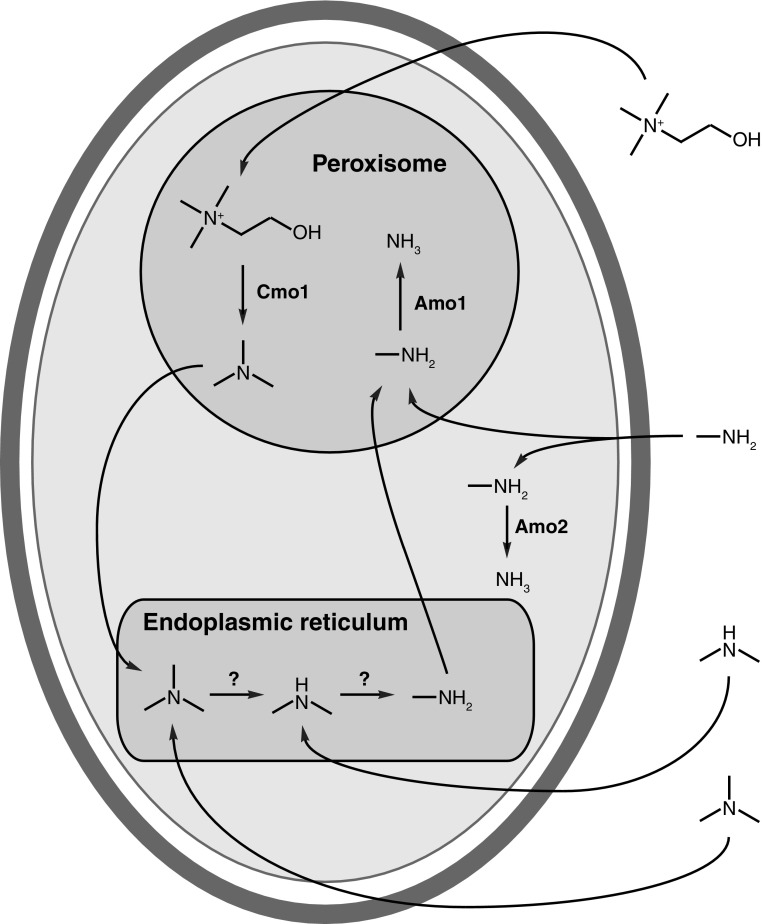
A model of intracellular compartmentalisation of catabolism of methylated amines in *Sc. stipitis* based on the data presented in this study as well as previous reports (Zwart et al. 1980, 1983; Green and Large 1984). In the proposed model, extracellular methylamine is demethylated by both Amo1 and Amo2 while methylamine produced through the catabolism of choline, trimethylamine or dimethylamine is predominantly demethylated by Amo1. The intracellular localisation of the Cmo1 choline monooxygenase has not yet been established and its placement in the peroxisome in the current diagram should be considered entirely speculative (see text for details)

The first general observation is that there appeared to be a consistent difference between growth patterns on methylamine versus dimethylamine, trimethylamine and choline in *Sc. stipitis*. Methylamine was the only methylated amine substrate where a growth defect was observed in a *Δamo2* strain (Fig. 2). Conversely, methylamine was the only methylated amine substrate where a growth defect was not observed in a *Δfgh1* strain (Fig. 3b) as well as the only methylated amine substrate where the growth dynamics of the *Δamo1* and *Δfgh1* strains deviated significantly from each other (Fig. S3). One hypothesis to explain this observation is that cell compartmentalisation distinguishes methylamine provided externally in the growth medium versus methylamine synthesised internally from the demethylation of dimethylamine, trimethylamine and choline (Fig. 5). External methylamine would be expected to enter directly into the cytosol with some portion further transported into the peroxisome. This could explain how both amine oxidase mutants displayed an extended lag before initiating growth (Fig. 2). Internally generated methylamine is expected to originate from the endoplasmic reticulum as this is the reported localisation of di- and trimethylamine monooxygenases in yeast (Green and Large 1983). The resulting methylamine is then transported into the peroxisome for the final demethylation step catalysed by Amo1. The cytosolic Amo2 would therefore play little or no role in the demethylation of internally generated methylamine in *Sc. stipitis* due to spatial separation between internally generated methylamine in the endoplasmic reticulum and the cytosolic Amo2 enzyme (Fig. 5). This scenario would require the majority of methylamine to be transferred from the endoplasmic reticulum to Amo1 in the peroxisome in a manner that would largely bypass Amo2 the cytosol. A simpler explanation is that the *AMO2* gene is expressed at a lower level in *Sc. stipitis* when cultivated on dimethylamine, trimethylamine or choline as compared to cultivation on methylamine. Previous enzyme activity studies on *O. boidinii* cell extracts showed that Amo2 enzymatic activity was not detectable when cells had been cultivated on dimethylamine, trimethylamine or choline (Haywood and Large 1981), which would support such a hypothesis. Expression analyses of individual genes in *Sc. stipitis* were beyond the scope of the current study but may be pursued in future studies. The intracellular localisation of the choline monooxygenase Cmo1 has not yet been established but the presence of a conserved C-terminus specific to fungal members of this protein family (Linder 2014) could indicate the presence of a non-canonical type 1 peroxisomal targeting signal (PTS1; Brocard and Hartig 2006). However, the current placement of Cmo1 in the peroxisome by the author as shown in Fig. 5 should be considered entirely speculative.

The second general observation is that budding yeasts appear to have at least one additional, as-yet unidentified enzyme capable of converting *S*-formylglutathione into glutathione (Fig. 3a). Weak but detectable growth in a *Δfgh1* background has now been demonstrated both in *Sc. stipitis* (this study; Fig. 3b) and in the methylotrophic yeast *O. boidinii* (Yurimoto et al. 2003). The fact that *SFA1* is still essential for growth on all tested methylated amines in *Sc. stipitis* (Fig. 3b) would argue against an entire pathway that is functionally redundant with the cyclic glutathione-dependent formaldehyde oxidation pathway in this yeast. However, this might not be the case in other yeasts such as *O. boidinii* where there is still detectable growth in a *Δsfa1* background (Yurimoto et al. 2003).

The third and final observation is that the demethylation of di- and trimethylamine does not appear to involve the CYP protein family in *Sc. stipitis*. Whether or not this also the case in other budding yeasts remains to be established. Previous studies on the catabolism of secondary and tertiary amines have established that these enzymatic activities are localised to the endoplasmic reticulum in yeast and are sensitive to heme-binding inhibitors such carbon monoxide and cyanide (Green and Large 1983, 1984; Fattakhova et al. 1991). One possibility is that a non-CYP family heme-containing enzyme catalyses the demethylation of di- and trimethylamine. The author has so far been unsuccessful in identifying any other likely gene candidates based on sequence similarity and gene annotation data alone. A more straightforward strategy to identify the genes encoding the enzymes responsible for these demethylation reactions would be comparative expression analysis of *Sc. stipitis* cultivated on di- or trimethylamine versus a non-methylated reference nitrogen substrate. The author would like to add that to the best of his knowledge, the deletion mutants of *PICST_49761* and *PICST_63000* generated in the present study represents the first attempt at phenotypic characterisation of CYP families CYP5217 and CYP501 in yeast.

In conclusion, the yeast *Sc. stipitis* has shown itself a promising system for the investigation of metabolic pathways not found in *Sa. cerevisiae*. The fairly recent development of genetic tools for targeted gene deletion in this species now enables more comprehensive reverse genetics studies in this yeast (Maassen et al. 2008; Linder 2014). As the present study demonstrates, there still remains much to be learned about the assimilation of alternative nitrogen sources in budding yeasts.

## Electronic supplementary material

Below is the link to the electronic supplementary material.

Supplementary Fig. S1 Design of the *Sc. stipitis* gene targeting constructs. a Targeting cassettes identical to the 5′ and 3′ flanking 500-bp regions (in reverse order) of each gene to be deleted were synthesised *de novo*, digested with EcoRI and HindIII (with the exception of the *Δpicst_65460* cassette, which was digested with EcoRI and StuI) and inserted into a pUC57 plasmid digested with the corresponding restriction enzymes. An in-frame stop codon (highlighted in bold font) would be added to the targeting cassette in those cases where the 5′ flanking sequence would include a portion of the 5′ coding sequence of the gene to be deleted. A short polylinker was incorporated at the 3′ end of each targeting construct to facilitate insertion of a selection marker. The restriction enzymes highlighted in red were used for the insertion of the BamHI/PstI-digested *DhHIS3* selection marker used in this study. b The *DhHIS3* selection marker was inserted into the short polylinker adjacent to the targeting cassette. c The finished integration construct. d The resulting pUC57-*DhHIS3*-*Δpicst_xxxxx* plasmid was linearised by digestion with SwaI, which enabled homologous recombination with *PICST_xxxxx* flanking regions (EPS 424 kb)

Supplementary Fig. S2 Control PCRs of the transgenic *Sc. stipitis* strains used in this study. The presence of a visible amplification product in the left panel indicates integration of the deletion construct at the correct genomic site. The absence of a visible amplification product in the right panel indicates the removal of the endogenous gene locus. Primer combinations for each of the PCR experiments are listed next to the corresponding gel image. Primer sequences are listed in Table S1 (EPS 9058 kb)

Supplementary Fig. S3 Comparison of growth phenotypes for genes *AMO1* and *FGH1* for the utilisation of methylated amines as sole nitrogen sources. *Sc. stipitis* strains TLSS001 (wildtype control), TLSS005 (*Δamo1*) and TLSS008 (*Δfgh1*) were cultured in 3 ml RSNLD medium supplemented with 10 mM of the indicated nitrogen source (initial OD_600_ 0.005). Samples were incubated in a shaker set at 30 °C, 200 r.p.m., and OD_600_ was measured after 6, 12 and 18 days. Growth assays were performed in triplicate with error bars indicating one standard deviation (EPS 332 kb)

Supplementary Fig. S4 Multiple sequence alignment of CYP monooxygenases in *O. parapolymorpha*, *Sa. cerevisiae* and *Sc. stipitis*. The alignment was formatted using BOXSHADE (http://www.ch.embnet.org/software/BOX_form.html) with 70% of residues having to agree at any position for shading. Sequences were retrieved from GenBank using the listed accession numbers with the exception of: (1) conceptual translation (translation Table 12) of NC_009048, residues 216,721–218,367; (2) conceptual translation (translation Table 12) of NC_009047, residues 1,074,603–1,076,360; (3) conceptual translation (translation Table 12) of NC_009047, residues 132,488–134,149 (reverse complement); (4) conceptual translation (translation Table 1) of NC_027864, residues 1,260,033–1,261,667 (reverse complement). Aligned positions used for the phylogenetic analysis in Fig. S5b are underlined in grey (EPS 2158 kb)

Supplementary Fig. S5 Identification of candidate CYP genes for the catabolism of di- and trimethylamine in *Sc. stipitis*. a *O. parapolymorpha* CBS 11,895 was cultured in 3 ml RSNLD medium supplemented with 10 mM of either sodium glutamate or trimethylamine as sole nitrogen sources (initial OD_600_ 0.005). Samples were incubated in a shaker set at 30 °C, 200 r.p.m., and OD_600_ was measured after 6, 12 and 18 days. Growth assays were performed in triplicate with error bars indicating one standard deviation. b Phylogenetic analysis of CYP genes in *O. parapolymorpha*, *Sa. cerevisiae* and *Sc. stipitis* based on 114 aligned amino acid positions (Fig. S4). Branch labels indicate the frequency of retained nodes among 10,000 bootstrap replicates. (EPS 350 kb)

Supplementary material 6 (DOC 33 kb)

## Abbreviations

CYP: Cytochrome P450 monooxygenase
IGR: Intergenic region
NHEJ: Non-homologous end-joining pathway
PTS: Peroxisomal targeting signal
