# Supplementary material for: Genetic redundancy in the catabolism of methylated amines in the yeast Scheffersomyces stipitis
Source: Antonie Van Leeuwenhoek. 2017 Oct 30;111(3):401–11. doi: 10.1007/s10482-017-0963-y (PMC5816127; doi:10.1007/s10482-017-0963-y)
Supplement: Supplementary file 6 — Supplementary material 6 (DOC 33 kb) [file 10482_2017_963_MOESM6_ESM.doc]

| **Supplementary Table S1** Control primers used in this study | |
| --- | --- |
| **Primer name** | **Sequence (5’ → 3’)** |
|  |  |
| pUC control fwd | TCGCCATTCAGGCTGCGCAACTGT |
| PICST_55523 ctrl fwd | TCGTAAGAGAGCACCATGGGCTT |
| PICST_55523 ctrl rev | TTCCCGTCATCAACATCTACACAT |
| PICST_83878 ctrl fwd | ATGCCTGCTAGACAGATGATCTT |
| PICST_83878 ctrl rev | TTGTCTCAGTGTATTGAGTGGTC |
| PICST_29252 ctrl fwd | ATTGGTCACTGGTAGAGTCTGGA |
| PICST_29252 ctrl rev | GATGACGAGAATGCAAGTGATGT |
| PICST_65460 ctrl fwd | ATATCGTAGAAGGCTATGATC |
| PICST_65460 ctrl rev | TGTGATGTGTTGATCAGTATG |
| PICST_49761 ctrl fwd | GGCCATCAATGAGATGTATACGT |
| PICST_49761 ctrl rev | ACATCTATCGAGAATGCCGTAGT |
| PICST_63000 ctrl fwd | AAGATATGTAGTGACTGGTGTGA |
| PICST_63000 ctrl rev | TCAAATCAACGGAGAGCCATGGA |
|  |  |
